# CT findings in sequel of COVID-19 pneumonia and its complications

**DOI:** 10.1259/bjro.20210055

**Published:** 2021-01-03

**Authors:** Mandeep Garg, Nidhi Prabhakar, Harsimran Bhatia, Sahajal Dhooria, Uma Debi, Valliapan Muthu, Muniraju Maralkunte, Inderpaul Sehgal, Ritesh Agarwal, MS Sandhu

**Affiliations:** 1 Department of Radiodiagnosis and Imaging, PGIMER, Chandigarh, India; 2 Department of Pulmonary Medicine, PGIMER, Chandigarh, India

## Abstract

A significant number of patients after initial recovery from COVID-19 continue to experience lingering symptoms of the disease that may last for weeks or even months. Lungs being the most commonly affected organ by COVID-19, bear the major brunt of the disease and thus it is imperative to be aware of the evolution of the pulmonary parenchymal changes over time. CT chest is the imaging modality of choice to evaluate post-COVID lungs. Persistent ground-glass opacities, septal thickening, parenchymal bands, crazy-paving, traction bronchiectasis and consolidation constitute the commonly encountered imaging patterns seen on CT in post-COVID-19 lungs. Few vulnerable patients can develop lung fibrosis and show honeycombing on CT. Additionally, many complications like superadded infections (bacterial and fungal), pulmonary thromboembolism and pseudoaneurysm formation are also being reported. In the present pictorial review, we have tried to show the entire CT spectrum of sequelae of COVID-19 pneumonia and commonly associated infections and vascular complications.

## Introduction

CT chest plays an indispensable role in the management of patients with coronavirus disease (COVID-19). Its role in acute COVID-19 infection has been extensively discussed and documented, while the data on the imaging features of the sequel of COVID-19 pneumonia and its complications is still emerging. A significant number of patients who recover from COVID-19 continue to experience its lingering symptoms even in their post-recovery phase, with cough and dyspnea being the typical respiratory symptoms.^1^ Thus, it is important to be aware of the persistent pulmonary parenchymal changes seen in the survivors of COVID-19 pneumonia and to know its evolution or resolution patterns over time. Additionally, it’s equally pertinent to be abreast of the imaging features of pulmonary complications, which are not uncommonly seen as immediate or long-term consequences of COVID-19.

## Discussion

### COVID-19 pneumonia sequel

**Figure 1.**
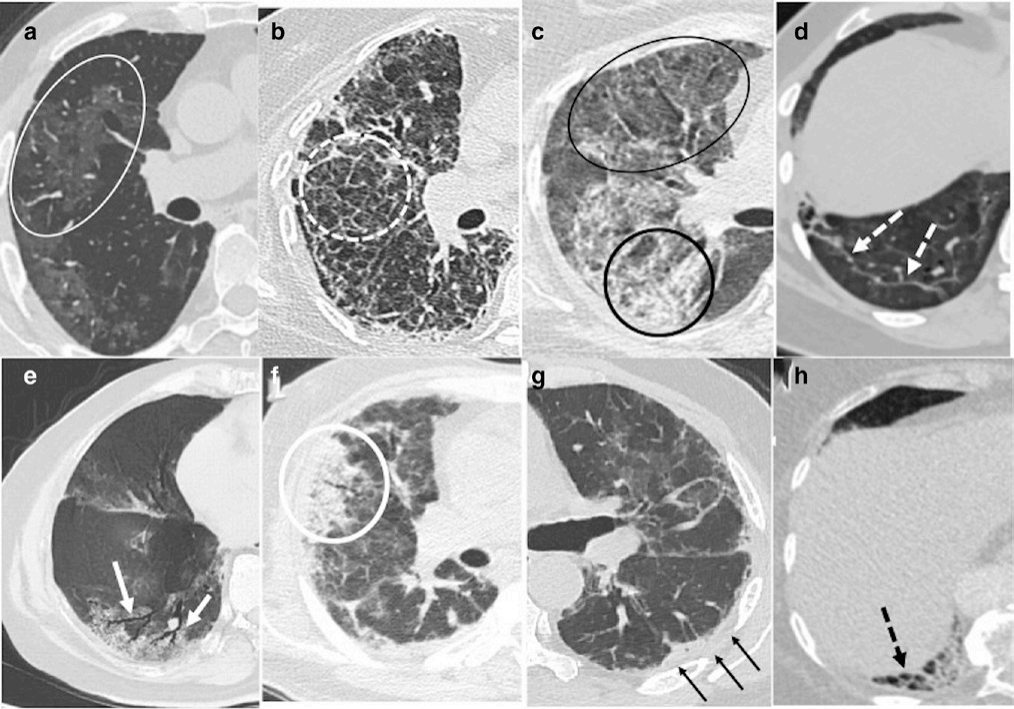
Spectrum of CT chest findings seen in post-COVID-19 lungs (a): Ground glass opacities (white oval) (b): Septal thickening (white dashed circle) (c): Crazy-paving (black oval and circle) (d): Parenchymal bands (white dashed arrows) (e): Traction bronchiectasis (white arrows) (f): Consolidation (white circle) (g): Pleural thickening (black arrows) and (h): Honeycombing (black dashed arrows).

**Figure 2.**
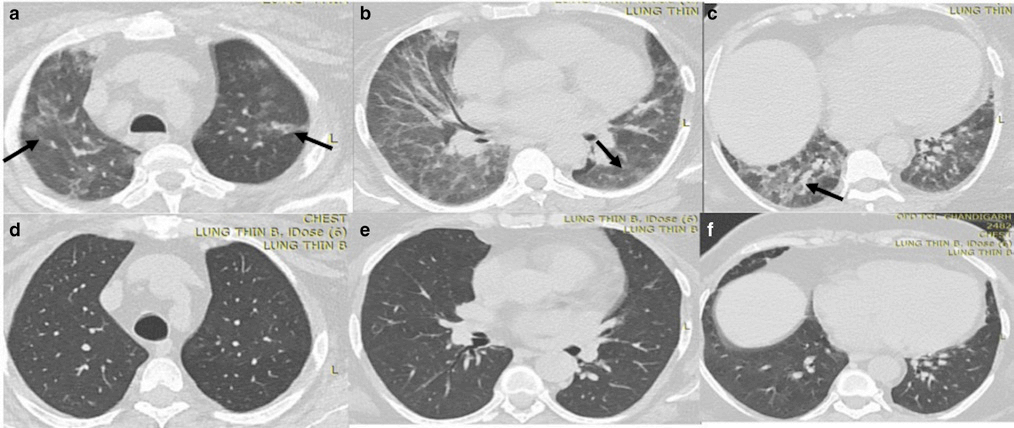
CT chest images of a 40-year-old female. (a–**c**): Axial CT Chest done during acute phase of COVID-19 pneumonia shows bilateral ground-glass opacities and interstitial septal thickening (black arrows). (**d–f**): Complete resolution is noted at 3 month follow-up scan.

**Figure 3.**
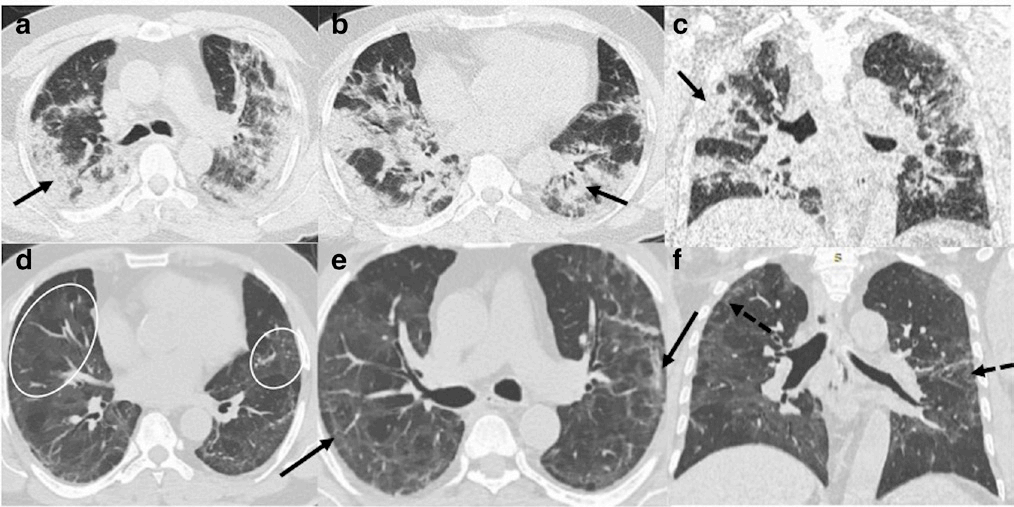
CT chest images of a 44-year-old male with persistent dyspnea showing residual abnormalities at 6 weeks after discharge from the hospital: (a–c) Axial and coronal sections of CT during acute phase showing consolidation with septal thickening predominantly in peripheral distribution (solid black arrows). (d–f) Axial and coronal CT sections at 6 weeks showing clearing of consolidative patches, but there are multiple ground-glass opacities (white circles), parenchymal bands and subpleural lines (black solid arrow) and reticulations in the bilateral lungs (dashed black arrow).

**Figure 4.**
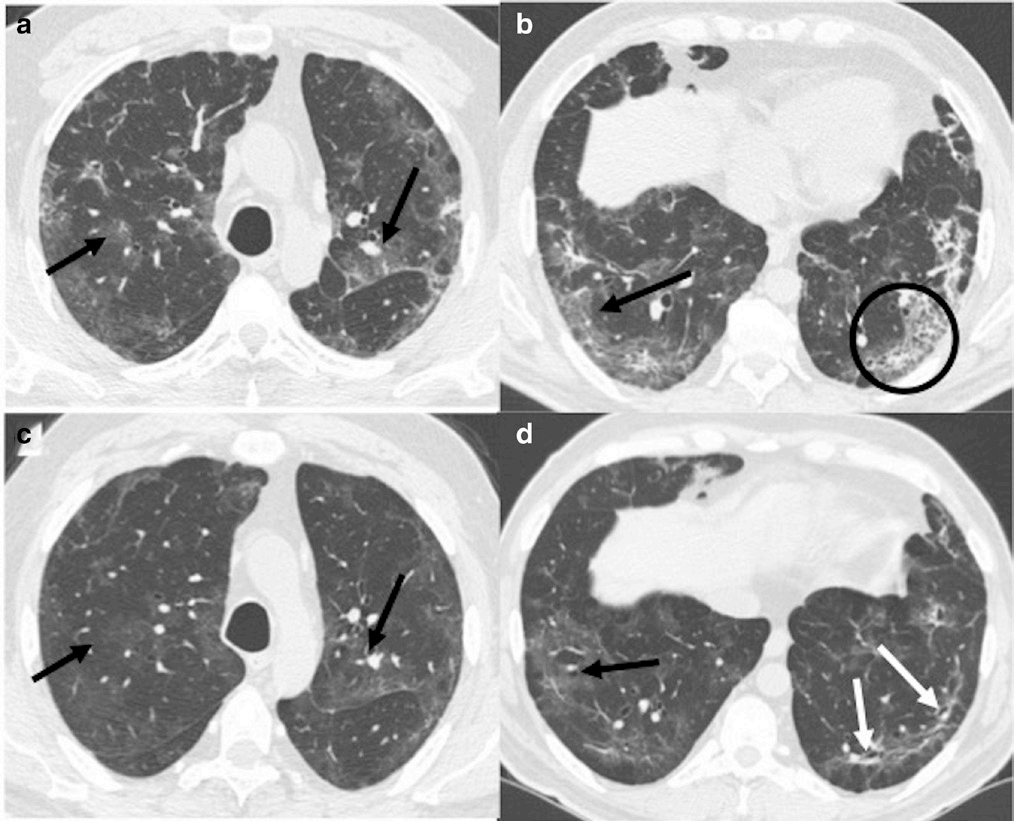
A 53-year-old male with severe COVID-19 pneumonia: (a**, b**) Axial CT chest sections, at the time of discharge from hospital, showing ground-glass opacities (black arrows) and extensive septal thickening with reticulations and mild traction bronchiectasis (white circle). (c**, d**) CT chest corresponding sections, 5 weeks later, showing ground-glass opacities and septal thickening have decreased with increased areas of traction bronchiectasis (white arrows).

**Figure 5.**
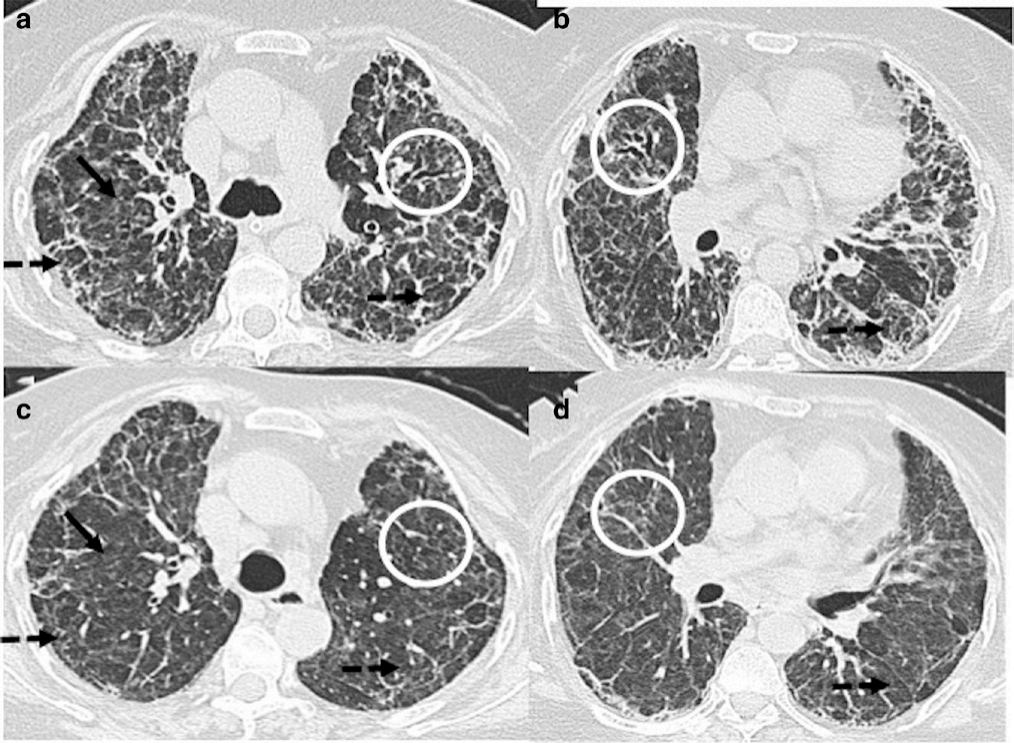
A 50-year-old female with COVID-19 pneumonia and Diabetes Mellitus: (a**, b**) Axial CT chest sections, at the time of discharge from hospital, showing mild ground-glass opacities (black arrows), extensive septal thickening (black dashed arrows) arrows), and few areas of traction bronchiectasis (white circles) (c**, d**) CT chest corresponding sections, 6 weeks later, showing ground-glass opacities and septal thickening have decreased with complete resolution of traction bronchiectasis.

High-resolution CT (HRCT) chest is considered the imaging modality of choice to evaluate the post-COVID-19 lung. Recently, some authors, in their follow-up studies, have tried to describe the evolution of pulmonary changes on CT scans. The common persistent pulmonary parenchymal abnormalities seen on CT scan^2–4^ at 3 months, 6 months, and 1 year follow-up include ground-glass opacities (GGOs), reticulations, septal thickening, crazy-paving, parenchymal bands, and traction bronchiectasis/bronchiolectasis; and rarely honeycombing (Figures 1–5).

Liu et al^5^ documented the CT findings on a 3 weeks follow-up CT and reported that about 50% of patients showed complete resolution of disease on CT. 40% of the patients, however, had persistent residual abnormalities on chest CT, of which fibrous stripes and GGOs were the most common. Zhao et al^2^ recruited 55 patients in a prospective study while determining the clinical characteristics/physiological features and imaging findings after 3 months of follow-up. They concluded that persistent HRCT abnormalities were found in a significant proportion of patients (about 70 %), with GGOs, interstitial thickening, and crazy-paving being the common imaging features.

In another prospective 6-month follow-up study conducted by Han et al^3^ on patients recovering from severe COVID-19, about 65% of patients showed complete resolution of the disease on CT. Residual ground-glass opacities or interstitial thickening were seen in about 27% of patients at 6 months follow-up.^3^ When compared to baseline imaging, few patients showed a decrease in density/attenuation while there was an apparent increase in the extent of opacity (described as the “tinted sign”).^5^ Fibrotic changes were documented in about 35% of patients at 6 months follow-up, and these included traction bronchiectasis, parenchymal bands, and/or honeycombing. The authors showed that patients who developed fibrotic lung disease at 6 months had a higher CT severity score at initial imaging. Also, this patient group had a higher incidence of acute respiratory distress syndrome (ARDS) during the acute phase and required frequent mechanical ventilation in comparison to the group that showed complete resolution or GGOs only (with no fibrotic changes). Han et al^4^ also followed up the patients who had lung sequelae at 6 months, till 1 year. Patients were classified into two groups, Group 1, which included patients with fibrotic interstitial lung abnormalities, while the second group, who had non-fibrotic interstitial lung abnormalities on the previous 6-month follow-up CT. 77% of patients in Group 1 had persistent fibrotic changes on CT even after 1 year, while 63% of patients in Group 2 showed complete resolution at 1 year.

Caruso et al^6^, in another prospective study on a large cohort of 118 patients recovered from moderate to severe COVID-19 pneumonia, showed that 72% of the patients had fibrotic-like changes at 6 month follow-up chest CT. The higher baseline lung severity score of >14 significantly predicted the presence of fibrotic-like changes in these patients in the post-recovery phase. The most common abnormality reported was persistent GGOs (in 42% of patients), with septal thickening and consolidation seen in 28 and 2% of patients, respectively.

### Complications of post-COVID-19 pneumonia

#### Fungal infections

The most commonly reported fungal infections in COVID-19 recovered patients are COVID-19 associated pulmonary aspergillosis (CAPA) and COVID-19 associated pulmonary mucormycosis (CAPM). Virus-induced immune dysregulation, underlying diabetes mellitus, and use of corticosteroids and other immunomodulatory drugs like tocilizumab create a favorable clinical setting in COVID-19 patients for superimposed fungal infections.^7^

**Figure 6.**
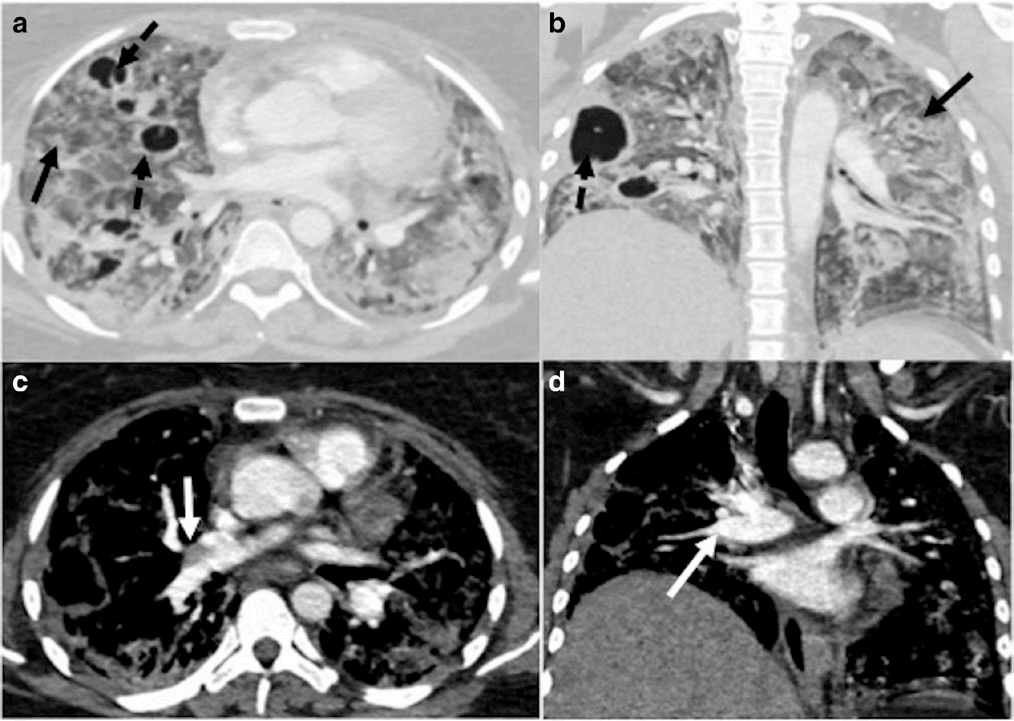
Contrast-enhanced CT in a patient of COVID-19 ARDS done at 4 weeks after his discharge from the hospital, showing features of invasive pulmonary aspergillosis. (a**, b**): Lung window sections showing diffuse ground-glass opacities in bilateral lungs (black arrows) with multiple cavitary lesions (black dashed arrows). (c**, d**): Mediastinal window sections showing eccentric partial filling defect suggestive of thrombus at the origin of the right lower lobe pulmonary artery. ARDS, acute respiratory distress syndrome.

**Figure 7.**
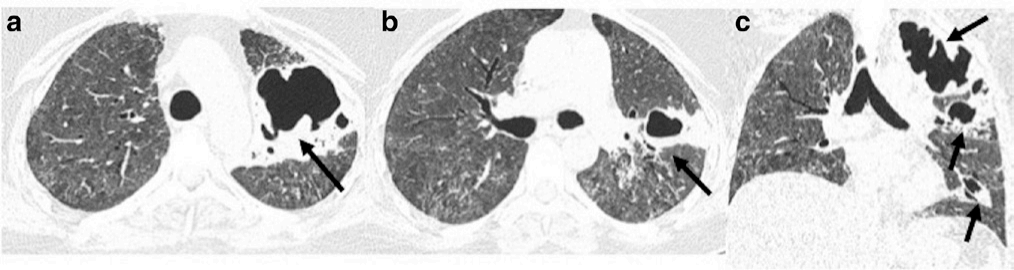
A 30-year-old male presented with new-onset fever and dyspnea 2 weeks after discharge; and was diagnosed as CAPM. (a–c) Lung window sections (axial and coronal) showing multiple cavitary lesions with irregular walls in the left lung (black arrows). CAPM, COVID-19 associated pulmonary mucormycosis.

**Figure 8.**
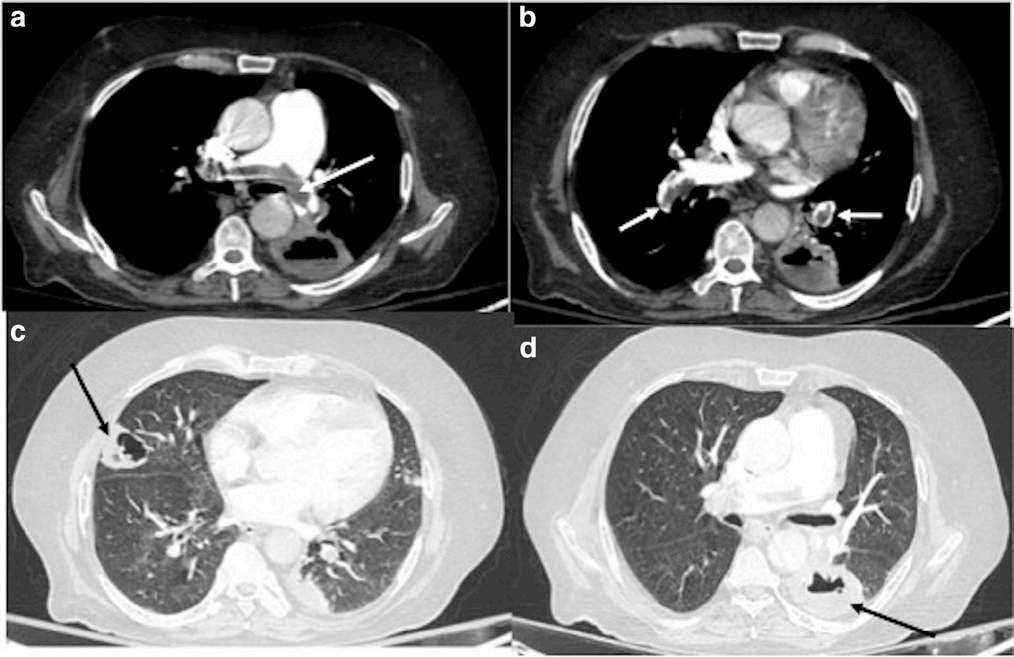
A 44-year-old male presented with sudden onset dyspnea, 3 weeks after recovering from COVID-19. (a**, b**): Axial CT pulmonary angiography sections show hypodense filling defect suggestive of acute thrombus within the main pulmonary artery extending into both lower lobe pulmonary arteries and segmental branches (white arrows); (c**, d**): Axial lung window sections showing cavitary lesions in both lungs (black arrows), which was later proven on sputum culture to be mucormycosis.

**Figure 9.**
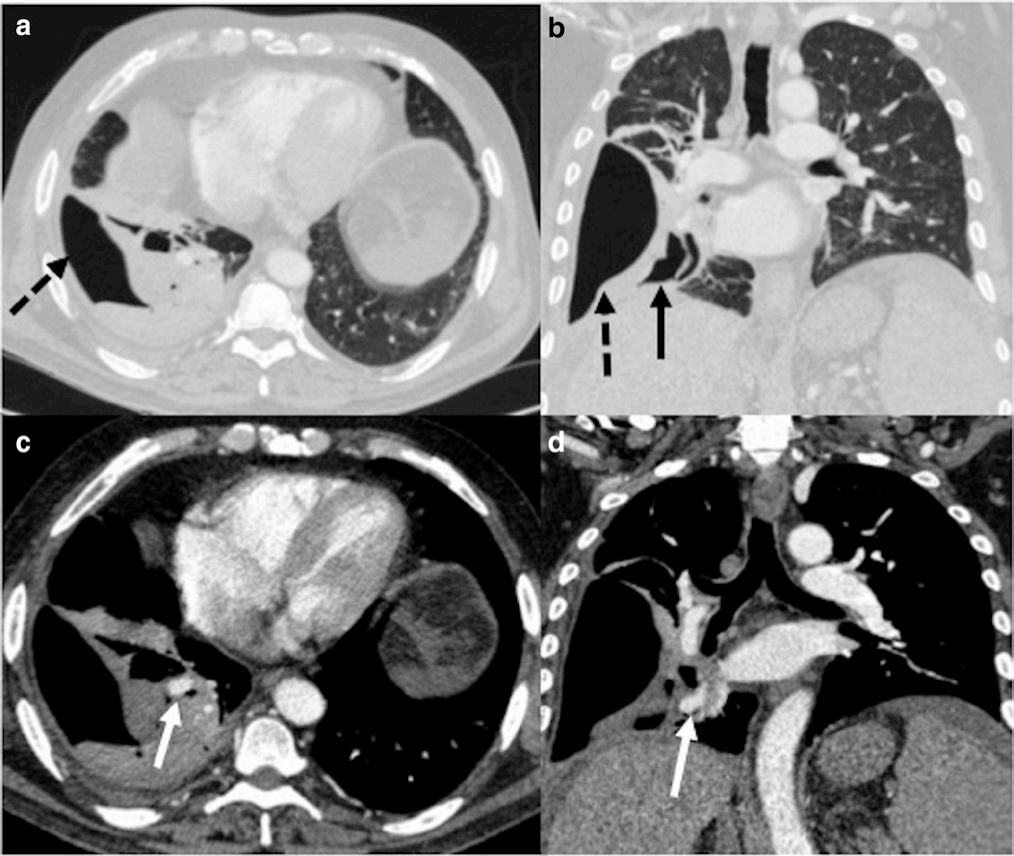
A 40-year-old male patient presented with hemoptysis 2 weeks after discharge from the hospital for COVID-19. (a**, b**) Lung window sections (axial and coronal) showing cavitary lesion in the right lung (black arrow) with loculated hydropneumothorax (black dashed arrows). (c**, d**) Mediastinal sections (axial and coronal) showing contrast-filled, saccular pseudoaneurysm in the cavity, arising from one of the segmental branches of the right lower lobe pulmonary artery (white arrows). KOH mount of sputum showed aseptate hyphae, and the patient was diagnosed as COVID-19 associated pulmonary mucormycosis with pseudoaneurysm.

The presence of cavitation, mass-like consolidation, pleural effusion, nodules, “halo sign”, “air crescent” sign, and “reverse halo” sign on the CT chest of COVID-19 recovered patients should alert the radiologist to the possible presence of fungal infections. The characteristic imaging features of invasive fungal infections on CT chest in patients without COVID-19 include peribronchial GGOs, lobar consolidation, nodules (<3 cm), mass-like consolidation (>3 cm), “halo sign”, “air crescent” sign, cavities, and “reverse halo” sign. However, some of these CT features like GGOs, consolidation, and “halo sign” can be overlapped by the changes of COVID-19 pneumonia sequel. The presence of “reverse halo” sign, pleural effusion, and concurrent sinus infection favors CAPM, whereas the presence of peribronchial consolidation, bronchial wall thickening, and clusters of centrilobular nodules point towards CAPA (Figures 6–8).^8^ However, in many instances, imaging alone may not suffice to differentiate various fungal pneumonias, and the laboratory/tissue diagnosis is needed for confirmation. Vascular complications like pseudoaneurysm have also been reported in patients with COVID-19 associated fungal infections (Figure 9).

#### Bacterial infections

**Figure 10.**
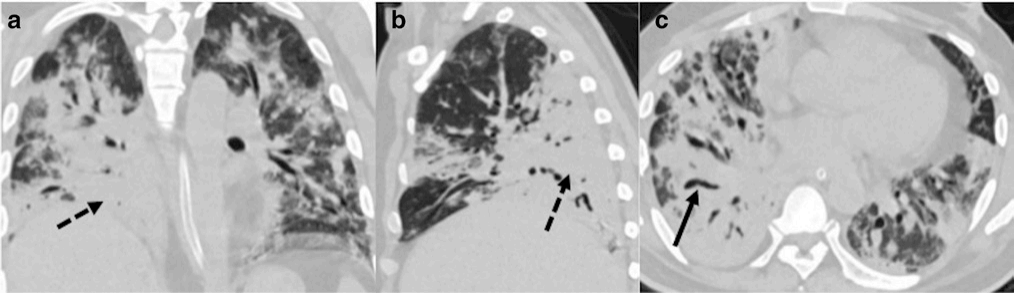
A 40-year-old male developed high grade fever, cough and expectoration after 6 weeks of testing negative for COVID-19. Sputum culture showed growth of *Streptococcus pneumoniae*: (a–c) Lung window sections showing multiple areas of lobar and peribronchial consolidation predominantly involving the right lower lobe (dashed black arrows), with presence of air bronchograms (solid black arrow).

**Figure 11.**
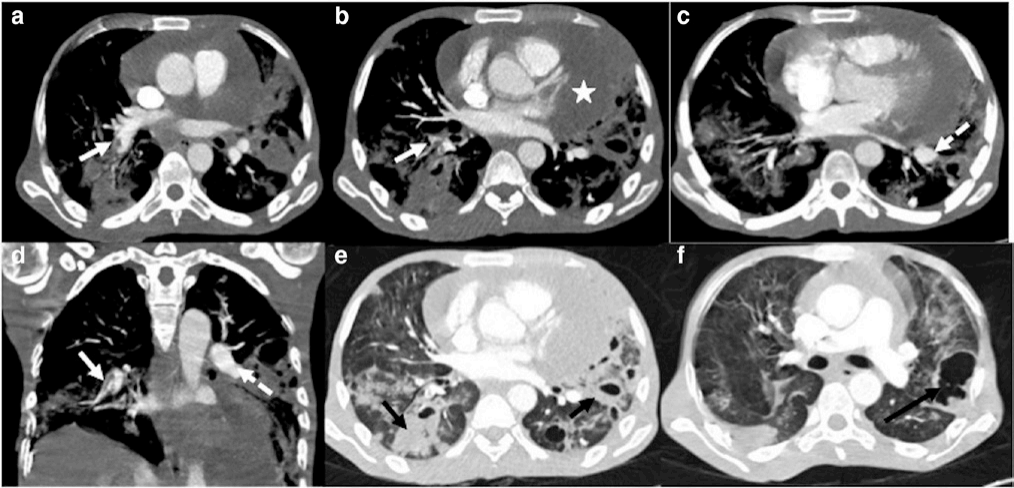
A 31-year-old male, a case of chronic kidney disease (on hemodialysis), complained of high grade fever and increasing dyspnea a week after recovering from COVID-19. The patient was diagnosed with infective endocarditis on echocardiography. CT chest images (**a–d**): Mediastinal sections showing thrombus in the right lower lobe pulmonary artery, extending into the segmental branches (white arrows); contrast filled pseudoaneurysm arising from the left lower lobe pulmonary artery (dashed white arrow). Moderate pericardial effusion is also noted (white asterisk). (**e, **f): Axial lung window sections showing multiple cavitary lesions in both lungs (black arrows). Blood culture revealed *Staphylococcus aureus*.

**Figure 12.**
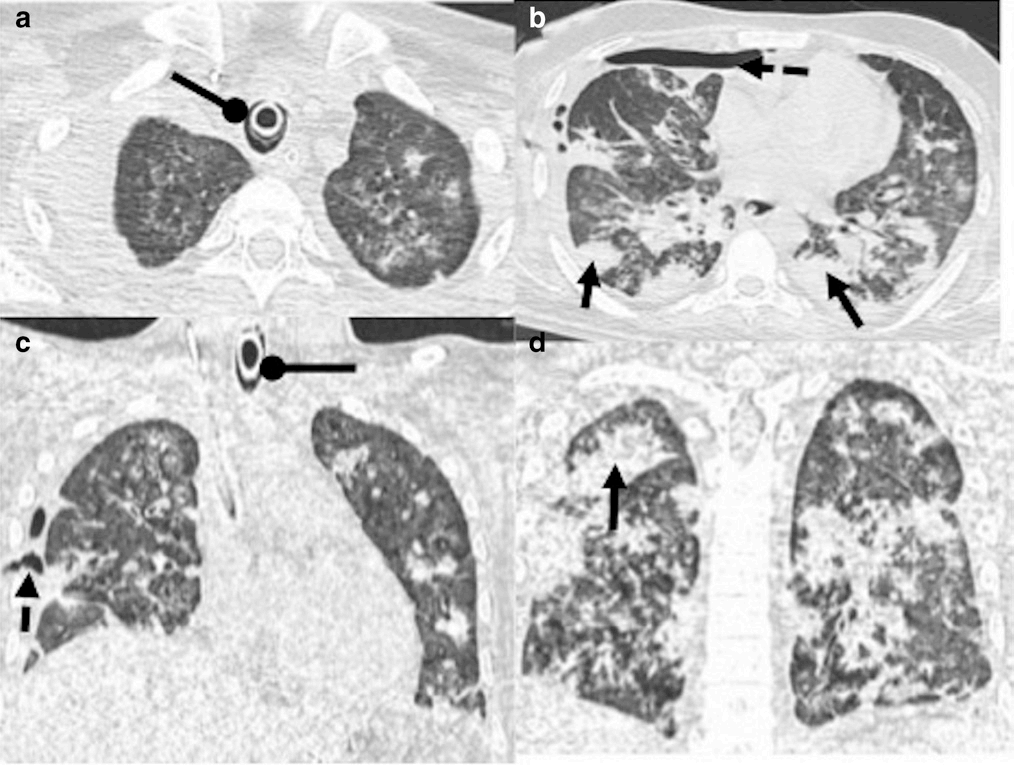
A 65-year-old male who was hospitalized for severe COVID-19 pneumonia and was on ventilator for more than a month. Patient subsequently developed fever again. While RT-PCR test for COVID-19 was negative, *Stenotrophomonas* was found on culture from endotracheal aspirate. Patient was diagnosed with ventilator associated pneumonia (a–d): Lung window sections showing patches of consolidation scattered in bilateral lung fields (black arrows) and minimal right-sided hydro-pneumothorax (dashed black arrows). Note is made of endotracheal tube *in situ* (round tip black arrows).

**Figure 13.**
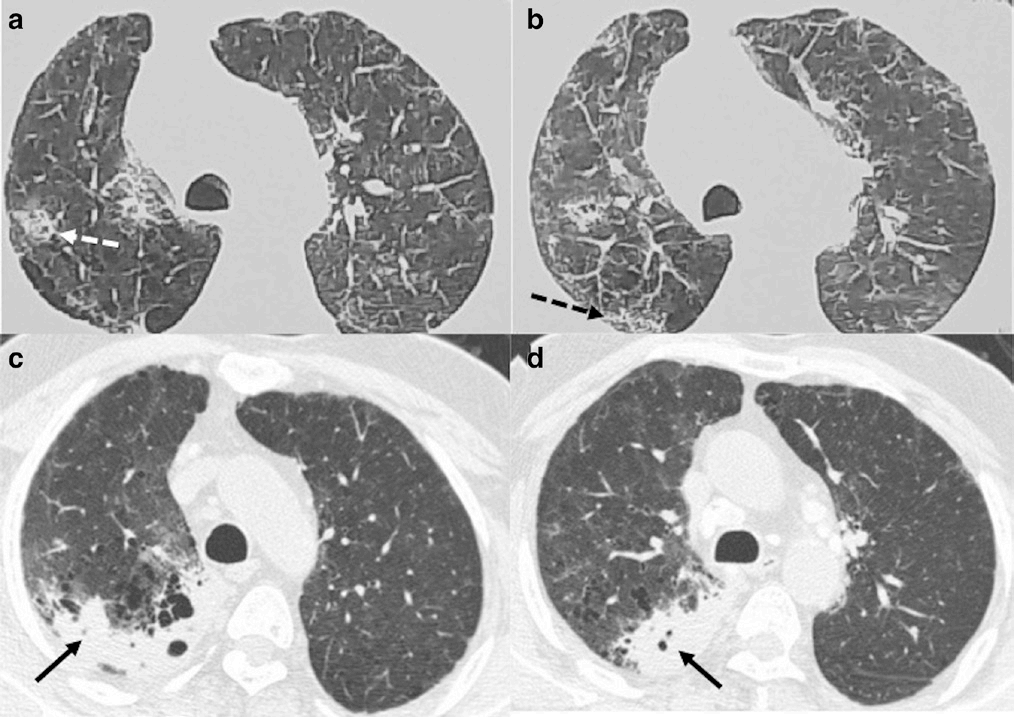
A 64-year-old male who was discharged from COVID hospital in an asymptomatic state, presented 7 weeks later with symptoms of cough and fever. (a**, b**): CT chest done at the time of discharge had few small areas of consolidation (dashed white arrow) and septal thickening (dashed black arrow). (c**, d**): CT chest done 7 weeks later showed increasing areas of consolidation with breakdown in right upper lobe (black arrows). FNAC done from the lesion showed acid-fast bacilli and positive culture for *Mycobacterium tuberculosis*.

COVID-19 patients are more vulnerable to develop superadded bacterial infections secondary to immunosuppression and immune dysregulation. In the published literature, bacterial co-infections during acute phase of covid-19 have been described with *Staphylococcus aureus, Streptococcus pneumoniae,* and *Hemophilus influenzae* being the most commonly isolated organisms.^9^ However, few vulnerable individuals remain prone to get these infections even in their post recovery phase (Figures 10 and 11). Due to the increased use of mechanical ventilation during this pandemic, ventilator-associated pneumonia (VAP) is another common occurrence. Maes et al^10^, in a retrospective study, concluded that the incidence of VAP was significantly higher in COVID-19 patients in comparison to the non-COVID-19 ICU patients on mechanical ventilation. The contributory factors that have been postulated for the increased incidence of bacterial infections include ARDS in the COVID-19 group, the requirement of ventilation for a longer duration, and the immunocompromised status of these patients (Figure 12). Pulmonary tuberculosis (TB) has also been reported in few patients following COVID-19 pneumonia (especially in TB endemic zones like India), primarily due to immunosuppression caused by steroids^11^ (Figure 13).

Identifying superimposed bacterial infections on imaging in post-COVID-19 pneumonia patients may be difficult because of the underlying lung parenchymal changes of COVID-19 sequelae. However, the presence of nodules, consolidation, and cavitation in the post-COVID-19 CT chest should raise the suspicion of superadded infections. Again, differentiating between bacterial and fungal pulmonary infections is difficult based on imaging alone. The presence of random nodules, “reverse halo” sign, cavitation, and concurrent sinus infection will favor the diagnosis of fungal infections, while the presence of lobar areas of consolidation, centrilobular nodules, and pleural effusion will favor the diagnosis of bacterial infections. However, cavitating bacterial pneumonias can be indistinguishable from fungal pneumonias, especially those caused by *Staphylococcus aureus*, *Klebsiella pneumoniae,* and *Mycobacterium tuberculosis*. The evaluation of sputum/endotracheal aspirate or bronchoalveolar lavage is necessary in many cases to arrive at the correct diagnosis.

### Pulmonary thromboembolism (PTE)

**Figure 14.**
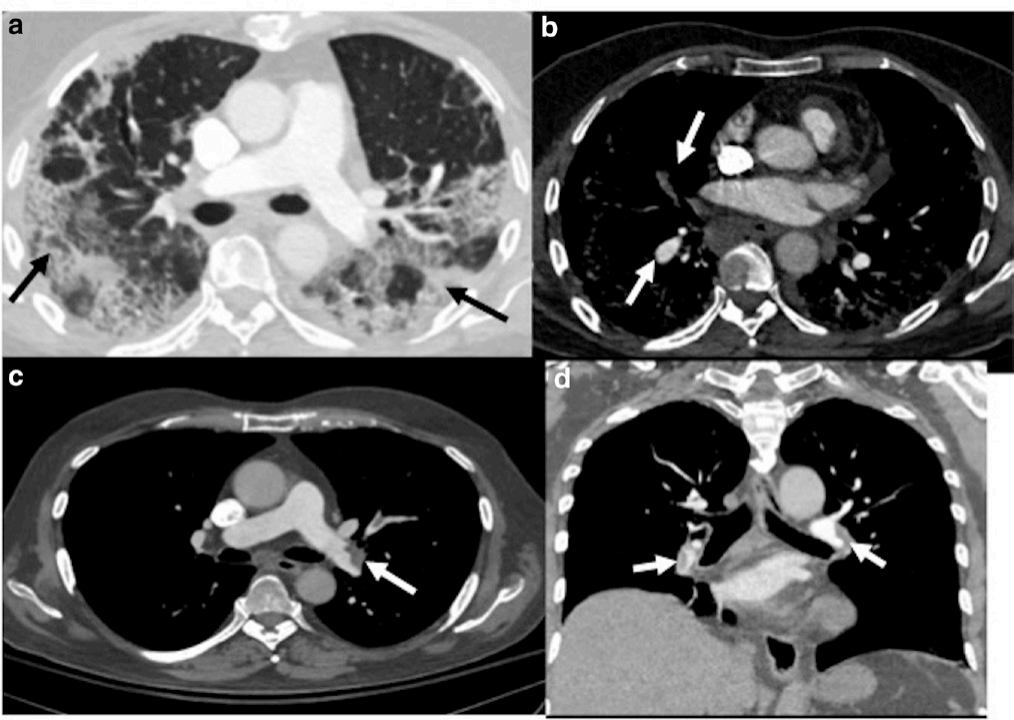
A 56-year-old male, recovered from COVID-19, presented with sudden onset of breathlessness, 20 days after hospital discharge. CT pulmonary angiography was done for the patient. (a): Axial lung window section showing septal thickening and ground-glass opacities involving bilateral lungs (black arrows). (b–d): Mediastinal window sections (axial and coronal) showing hypodense filling defects suggestive of thrombi in left main pulmonary artery, anterior segmental branch of right upper lobe pulmonary artery and right lower lobe pulmonary artery (white arrows).

COVID-19 is a pro-coagulant disease due to the cytokine storm, endothelitis, and localized pulmonary microangiopathic initiated by the virus in the body that predisposes to venous thromboembolism. Many studies have reported the increased risk of pulmonary thromboembolism in acute COVID-19 patients. Vlachou et al^12^ found that the risk of thrombosis in pulmonary vessels continues even in the post-recovery phase at least up to 4 weeks. CT pulmonary angiography (CTPA) is the modality of choice to evaluate patients with suspected thromboembolism. Acute PTE is characterized by a complete or partial intraluminal filling defect seen in the vessel lumen (Figure 14).

### Pneumothorax

**Figure 15.**
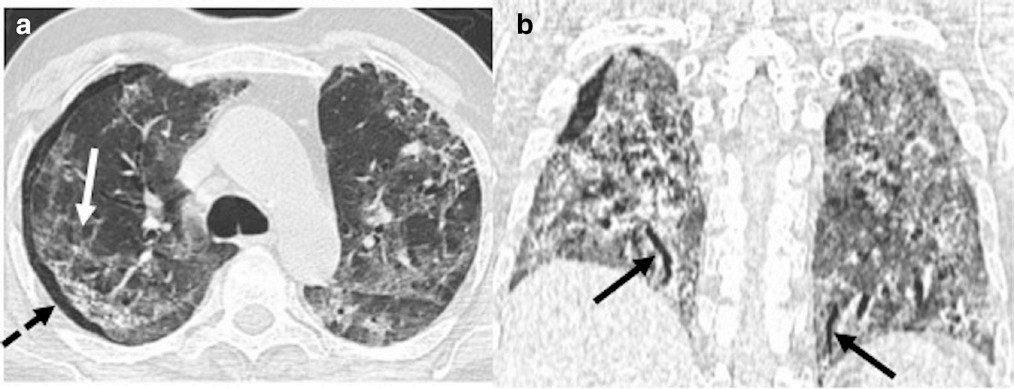
A 50-year-old male patient presented with new-onset dyspnea 4 weeks after discharge from the hospital and testing negative for COVID-19. (**a, b**): CT lung window axial and coronal sections showing ground-glass opacities and septal thickening involving bilateral lungs (white arrows) with right pneumothorax (dashed black arrow). Traction bronchiectasis (black arrows) is noted in bilateral lungs.

Pneumothorax has been frequently reported in patients of COVID-19 and can be associated with concurrent pneumomediastinum. Although the reported incidence of barotrauma-induced pneumothorax has been reported to be high in mechanically ventilated patients of COVID-19 pneumonia, all patients developing pneumomediastinum and/or pneumothorax are not mechanically ventilated. Sihoe et al^13^ reported spontaneous pneumothorax in hospitalized patients of COVID-19 with an incidence of 1.7%. Recently, there have been many reported cases of spontaneous pneumothorax in patients who have recovered from COVID-19 pneumonia.^14,15^ Possible causative factors include the presence of lung cysts/bullae and ischemic/inflammatory lung parenchymal damage caused by the virus (Figure 15).

## Conclusion

CT chest has been widely used in patients for the diagnosis and management of acute COVID-19. Patients after recovery from the viral illness may present with a myriad of clinical symptoms or complications. CT chest is an essential diagnostic tool in the post-COVID-19 recovery period to look for resolution/evolution of lung disease, detect its sequelae and identify complications. Knowledge of the imaging spectrum of post-COVID-19 lung and associated complications will help the radiologist diagnose the patient accurately, thus allowing appropriate and prompt treatment in this patient group.
